# Isoflurane Damages the Developing Brain of Mice and Induces Subsequent Learning and Memory Deficits through FASL-FAS Signaling

**DOI:** 10.1155/2015/315872

**Published:** 2015-11-01

**Authors:** Xiuwen Yi, Yirong Cai, Wenxian Li

**Affiliations:** Department of Anesthesiology, The Eye, Ear, Nose and Throat Hospital, Fudan University, Shanghai 200031, China

## Abstract

*Background*. Isoflurane disrupts brain development of neonatal mice, but its mechanism is unclear. We explored whether isoflurane damaged developing hippocampi through FASL-FAS signaling pathway, which is a well-known pathway of apoptosis. *Method*. Wild type and FAS- or FASL-gene-knockout mice aged 7 days were exposed to either isoflurane or pure oxygen. We used western blotting to study expressions of caspase-3, FAS (CD95), and FAS ligand (FASL or CD95L) proteins, TUNEL staining to count apoptotic cells in hippocampus, and Morris water maze (MWM) to evaluate learning and memory. *Result*. Isoflurane increased expression of FAS and FASL proteins in wild type mice. Compared to isoflurane-treated FAS- and FASL-knockout mice, isoflurane-treated wild type mice had higher expression of caspase-3 and more TUNEL-positive hippocampal cells. Expression of caspase-3 in wild isoflurane group, wild control group, FAS/FASL-gene-knockout control group, and FAS/FASL-gene-knockout isoflurane group showed FAS or FASL gene knockout might attenuate increase of caspase-3 caused by isoflurane. MWM showed isoflurane treatment of wild type mice significantly prolonged escape latency and reduced platform crossing times compared with gene-knockout isoflurane-treated groups. *Conclusion*. Isoflurane induces apoptosis in developing hippocampi of wild type mice but not in FAS- and FASL-knockout mice and damages brain development through FASL-FAS signaling.

## 1. Introduction

Isoflurane is a commonly used clinical anesthetic because it is fast-acting, produces little irritability, and stabilizes hemodynamic status of patients. It is also a convenient inhaled anesthetic for children. General anesthesia with isoflurane for children, ranging from babies to adolescents, is a common practice in modern anesthesiology for surgical or procedural pain relief [1].

Learning and memory ability depends on structures within the medial temporal lobe, including the hippocampal region (subicular complex, CA fields, and dentate gyrus) and the adjacent perirhinal, entorhinal, and parahippocampal cortices [2]. The development of neurons in the hippocampus is closely associated with the learning and memory ability of the developing brain. In recent studies, researchers showed many adverse effects caused by isoflurane on the developing brain especially in the embryonic period, neonatal period, and infancy. They concluded that isoflurane could increase neurodegeneration in the developing hippocampus of fetal rats [3]. Moreover, commonly used inhaled anesthetics including isoflurane, sevoflurane, and desflurane demonstrated similar neurotoxic profiles in neonatal mice, suggesting that developmental neurotoxicity is a common feature of all inhaled anesthetics [4]. In the developing nonhuman primate brain, isoflurane induces neuronal apoptosis in neonatal rhesus macaques [5]. General anesthesia, including isoflurane, caused permanent neuronal deletion in the most vulnerable brain regions including the hippocampus in 7-day-old rat pups [1]. Interestingly, treating pregnant rats with isoflurane caused spatial memory and learning impairments and greater neurodegeneration in the offspring rats compared with control groups [6]. However, the detailed mechanisms underlying the deleterious effect of isoflurane on brain development are still largely unclear. Some evidence indicates that isoflurane targets the enzymes GAD65 and GAD67 in the developing nervous system, suggesting that isoflurane inhibits the developing nervous system [7]. Another line of evidence revealed that isoflurane induces long-term neurobehavioral consequences through damage of oligodendrocytes [8]. Inhaled anesthetics may also alter hippocampal synaptic plasticity to affect the development and wiring of the rat brain [9]. Here, we study another possible molecular mechanism involving the FASL-FAS signaling pathway.

The FAS singling pathway is a common way to induce apoptosis in cells. The FAS receptor protein is also known as APO-1 or CD95. It belongs to the subgroup of the tumor necrosis factor receptor (TNF-R) family that can trigger apoptosis. Its physiological ligand, FASL (CD95L), is a member of the corresponding TNF cytokine family. FAS and FASL play critical roles in the immune system, in particular in the killing of pathogen-infected target cells and the death of no longer needed, potentially deleterious as well as autoreactive lymphocytes [10]. Further, the FAS signaling machinery can also be involved in nonapoptotic processes, including cellular activation, differentiation, and proliferation [11]. Various drugs and substances can induce neuronal apoptosis through FASL-FAS signaling and may involve GSK-3 inhibition [12]. For instance, the commonly used GSK-3 inhibitor, lithium, reportedly facilitates apoptotic signaling induced by the activation of the FAS death domain-containing receptor [13]. While phenylalanine is an essential amino acid for humans, cytotoxic levels of phenylalanine can induce apoptosis in cultured cortical neurons through FASL-FAS signaling [14]. Therefore, FASL-FAS signaling is a major regulator of apoptosis; whether isoflurane can induce apoptosis through this pathway is unknown.

To begin investigating this issue, we treated primary cortical neurons with isoflurane and performed a microarray analysis. We found that isoflurane upregulated some genes coding apoptosis-related proteins and downregulated the genes coding proteins involved in learning and memory. Importantly, we found that FASL-FAS signaling was activated in primary cortical neurons by isoflurane, while the levels of FASL and FAS proteins were increased. Based on these preliminary findings, we further investigated the effects of isoflurane on the development of the intact hippocampus in neonatal mice through the FASL-FAS pathway, as well as how hippocampal learning is affected by isoflurane.

## 2. Materials and Method

### 2.1. Animal Preparation

The Central Laboratory and Center of Animal Experiments of the Eye, Ear, Nose and Throat Hospital at Fudan University provided most of the experimental procedures and protocols used in this study. All efforts were made to minimize the number of animals used and their suffering. Seven-day-old C57BL6/J mice together with their mothers (SLAC Laboratory Animal, Shanghai) and 7-day-old B6.MRL-Fas^lpr/NJU^ and B6Smn.C3-Fasl^gld/NJU^ C57 mice together with their mothers (Institute for Biological and Medicine Studies of Nanjing University, Nanjing) were housed in clean cages and the room temperature was maintained at 24 ± 1°C, with a 12-h light-dark cycle. We used neonatal mice aged 7 days to examine apoptosis and mice aged 25 days to examine the learning and memory. We chose these two ages because previous studies demonstrated that 7-day-old mice exhibit robust anesthesia-induced cell death, with mortality lower than 20% during 6-h anesthetic exposure [15], and mice at 25 days of age are the youngest able to undergo the Morris water maze test.

### 2.2. Anesthesia Exposure

Wild type C57BL/6 mice, FAS-knockout mice, and FASL-knockout mice aged 7 days were randomly assigned to two groups, treated with either control pure oxygen or isoflurane. In total, there were six groups: wild type isoflurane group, wild type pure oxygen group (wild type control group), FAS-knockout pure oxygen group (FAS-knockout control group), FAS-knockout isoflurane group, FASL-knockout pure oxygen group (FASL-knockout control group), and FASL-knockout isoflurane group. Isoflurane groups received 1.5% isoflurane in 100% oxygen for 2 h per day for a total of 3 days. Control groups received only 100% oxygen for the same period as the isoflurane groups. Temperature and humidity were kept steady. Pups were fed by their mothers daily after anesthesia. At postnatal day (P) 9, mice were killed and the hippocampi were used to detect the apoptotic factor, caspase-3, by western blot. Mice that were not sacrificed at P9 were kept until P25 for the Morris water maze test. Anesthesia with 1.5% isoflurane for 2 h in neonatal mice did not cause significant changes in blood pressure and blood gas showed in previous study [16].

### 2.3. Tissue Preparation

Mice were killed and the brains were immediately harvested, and hippocampi were dissected for western blot analysis. The harvested hippocampus tissues were homogenized on ice using radioimmunoprecipitation assay (50 mM Tris, pH 7.4, 150 mM NaCl, 1% NP-40, 0.5% sodium deoxycholate, and 0.1% SDS) plus protease inhibitors (1 mM PMSF). The lysates were collected, centrifuged at 12,000 rpm for 5 min at 4°C, and quantified for total protein with the enhanced BCA protein assay kit (P0010S, Beyotime Institute of Biotechnology, China).

Another group of pups were perfused transcardially with heparinized saline followed by 4% paraformaldehyde in 0.1 M phosphate buffer (pH 7.4). The brains were then removed and postfixed overnight in the same fixative at 4°C for 24 h. Thereafter, brains were frozen at −20°C and stored at −80°C until use. Serial coronal sections (10 *μ*m) were cut on a cryostat (CM3050 S, Leica Biosystems, Germany), mounted on coverslips, and stored at −80°C. Coronal brain sections were studied for apoptosis by TUNEL staining. The other mice were fed to 25 days of age to test their learning and memory ability through Morris water maze.

### 2.4. Microarray Analysis

Microarray data were obtained from publicly available Gene Expression Omnibus (GEO) databases. Dataset GSE2982 was chosen as it fulfilled the requirements for our study. To investigate FAS signaling pathways that were dysregulated in isoflurane-treated neurons, gene set enrichment analysis (GSEA) was performed, following the developer's protocol (http://www.broad.mit.edu/gsea/). Gene sets are available from the Molecular Signatures Database (MolSigDB, http://www.broad.mit.edu/gsea/msigdb/msigdb_index.html). Briefly, normalized microarray expression data were ranked/ordered by differential expression. Normalized microarray expression data were expressed in log_2_ scale. Up- or downregulated genes were selected based on both a change in expression by 1.5 or −1.5 and statistical significance (*P* < 0.05) via Student's *t*-test. Default parameters were used. The phenotype was permutated 1,000 times. Heat maps of the expression data were generated by color-coding gene expression intensities. The color code shows higher or lower expression relative to the mean expression of that gene in all samples.

### 2.5. Western Blot Analysis for FAS, FASL, and Caspase-3

The hippocampi were harvested at the end of the anesthesia and were subjected to western blot analysis using the methods described by Kim et al. [17]. Briefly, protein samples were quantitated by the enhanced BCA protein assay kit (P0010S, Beyotime Institute of Biotechnology, China). SDS-PAGE was carried out using 10–12% gradient Tris/glycine gels under reducing conditions. Proteins were transferred to a polyvinylidene difluoride membrane (Bio-Rad) using a semidry electrotransfer system (Bio-Rad 043BR39099). The blots were blocked with 5% nonfat dry milk in TBST (25 mM Tris (pH 7.6), 137 mM NaCl, and 0.1% Tween-20) for 1.5 h and incubated with primary antibodies (caspase-3 antibody, 1 : 1,000, Abcam ab47131; CD95 antibody, 1 : 1,000, Bioworld BS1461; CD95 ligand antibody, 1 : 1,500, Bioworld BS1122; *β*-actin antibody, 1 : 5,000, Bioworld BS6007M) overnight at 4°C and subsequently with secondary antibodies (horseradish peroxidase-conjugated goat anti-mouse or anti-rabbit antibodies, 1 : 5,000, Bioworld BS12478 or Bioworld BS13278) in TBST plus 5% nonfat dry milk. The caspase-3 antibody was used to recognize the caspase-3 fragment (17–20 kDa), which results from cleavage at aspartate 175, and FL-caspase-3 (35–40 kDa). The antibody to the nontargeted protein *β*-actin (42 kDa) was used to control for loading differences in total protein amounts. Between steps, the blots were washed with TBST for 20 min. The blot was visualized using the Kodak image station (Kodak 4000MM PRO, USA). Each band in the blot represents an independent experiment. We averaged the results from 3–6 independent experiments.

### 2.6. TUNEL Staining for DNA Fragmentation

Mouse brains were removed and kept at 4°C in 4% paraformaldehyde. Serial coronal sections (10 *μ*m) were cut on a cryostat (CM3050 S, Leica Biosystems, Germany) and mounted on coverslips. Sections were permeabilized with proteinase K solution (20 g/mL) for 20 min. Terminal deoxynucleotidyl transferase (TdT) and dUTP (11684817910, Roche) were added to the sections and incubated in a humidified chamber at 37°C for 2 h. The reaction was then stopped and followed by colorization with DAPI (C1002, Beyotime, China) for 10 min. Then, coverslips were mounted on glass slides with antifade mounting medium (P0126, Beyotime, China). Finally, the sections were analyzed under a light microscope (ECLIPSE TI-SR, NIKON, Japan) with 5x and 20x objective lenses and photographs of the sections were taken. An investigator who was blind to the experimental design counted the number of TUNEL-positive cells using Image LAS V4.1 (Leica Microsystems, Switzerland).

### 2.7. Morris Water Maze (MWM)

A round, fiberglass pool, 150 cm in diameter and 60 cm in height, was filled with water to a height of 1.5-cm above the top of the movable clear 15 cm diameter platform. The pool was located in a room with numerous visual cues (including computers, posters, and desks) that remained constant during the studies. Water was kept at 20°C and opacified with titanium dioxide throughout all training and testing. A video tracking system recorded the swimming motions of animals and the data were analyzed using motion-detection software for the MWM (Actimetrics software, Evanston, IL, USA). After every trial, each rat was placed in a holding cage, under an infrared heat lamp, before returning to its regular cage.

P25 mice were tested in the MWM 4 times per day for 5 days. Each mouse was placed in the pool to search for the platform at a random starting point. Mice that found the platform were allowed to stay on it for 15 s. The time taken to find the hidden platform was recorded as escape latency. If a mouse did not find the platform within a 90 s period, it was gently guided to the platform and allowed to stay on it for 15 s. The video tracking system recorded the swimming motions of the animals, and the data were analyzed using motion-detection software for the MWM (Institute of Materia Medica, Medical College of Tongji University, Shanghai, China). At the end of the reference training (P29), the platform was removed from the pool and the mouse was placed in the opposite quadrant. Each mouse was allowed to swim for 90 s, and the number of times the mouse swam across the platform area was recorded as platform crossing times. After every trial, each mouse was placed in a holding cage under a heat lamp for 1-2 min to dry before being returned to its regular cage.

### 2.8. Statistical Analysis

Data regarding biochemical changes and escape latency are expressed as means ± SD. The data for platform crossing time were not distributed normally and thus are expressed as medians and interquartile ranges. The number of samples varied from 5 to 10, and the samples were distributed normally with the exception of platform crossing time (tested by normality test, data not shown). A two-sample *t*-test was used to determine the difference between the anesthesia and control groups in levels of FAS, FASL, caspase-3, and TUNEL-positive cells. A two-way ANOVA with repeated measurements was used to analyze the difference of caspase-3 levels between the anesthesia and control groups. Interaction between time and group factors in a two-way ANOVA was used to analyze the difference of escape latency between mice in the control group and mice treated with anesthesia in the MWM. The Mann-Whitney *U* test was used to detect the difference of platform crossing times between the isoflurane treatment and control conditions. There were no missing data for the variables of MWM (escape latency and platform crossing time) during the data analysis. In all experiments, differences were considered statistically significant at *P* < 0.05. GraphPad Prism software version 5.01 (GraphPad Software Inc., California, USA) was used to analyze the data.

## 3. Results

### 3.1. Genome-Wide mRNA Profiling of Primary Cortical Neurons Treated with Isoflurane

**Figure 1:**
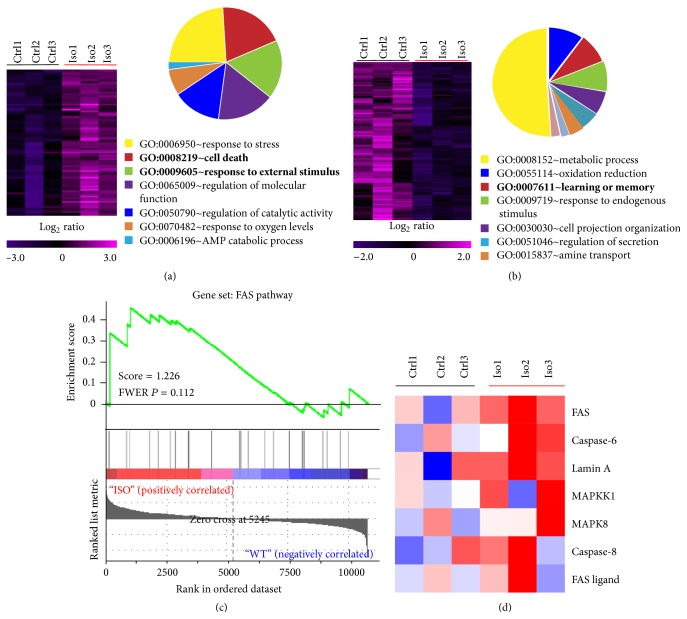
(a, b) Heat map of significantly upregulated genes (a) or downregulated genes (b) in primary cortical neurons treated with isoflurane. Diagrams depicting differentially expressed genes were grouped into distinct categories based on the function of encoded proteins shown on the left. (c) Quantitative pathway analysis using gene set enrichment showed the enrichment of FAS signaling in isoflurane-treated primary cortical neurons. (d) Heat map of microarray analysis demonstrated that the FAS signaling related transcripts were upregulated, including FAS and its ligand, FASL.

We first investigated the possible connection among isoflurane treatment, FASL-FAS signaling, and neurotoxicity. In primary cortical neurons, isoflurane upregulated the expression of genes like apoptosis-related genes and downregulated the expression of genes involved in learning and memory (Figures 1(a) and 1(b)). The activity of FASL-FAS signaling was verified through quantitative pathway analysis and the expression of proteins related to the signaling pathway was dramatically increased (Figures 1(c) and 1(d)).

### 3.2. Isoflurane Increases the Expression of FAS, FASL, and Caspase-3 in Wild Type Neonatal Hippocampus

**Figure 2:**
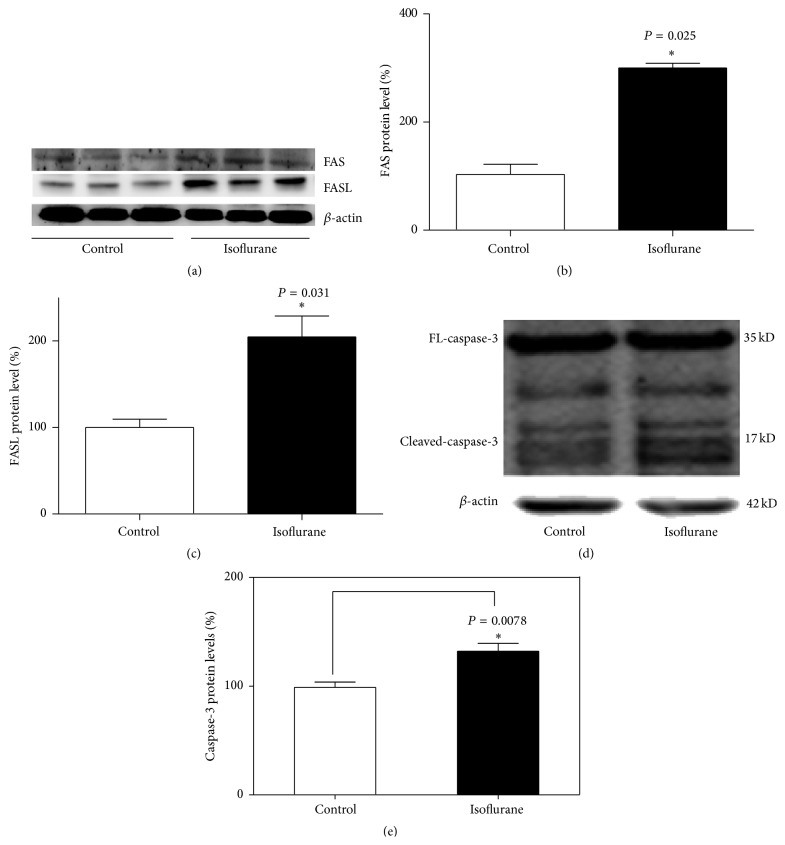
(a) Western blot showing the expression of FAS and FASL proteins in the hippocampus was increased by isoflurane compared with control group (*n* = 5). (b) Quantification of the western blot showed that isoflurane increased the expression of FAS protein in the mouse hippocampus compared to the wild control group. (c) Quantification of the western blot showed that isoflurane increased the expression of FASL protein in the mouse hippocampus compared to the wild control group. (d) Isoflurane increased the level of caspase-3 compared with the wild control group (*n* = 5). (e) Quantification of the western blot showed that isoflurane increased the expression of caspase-3 protein in the mouse hippocampus compared to the wild control group. Error bars represent the standard deviation (SD) of two independent experiments; ^*∗*^ *P* < 0.05.

We determined the degree of apoptosis by detecting the expression of caspase-3 in the hippocampus of wild type neonatal mouse after isoflurane treatment. We also measured the activation of the FASL-FAS pathway by detecting the expression of FAS and FASL proteins in the hippocampus. After treating P7 mice with isoflurane for 2 h per day for 3 days, wild type neonatal mouse showed large increases in the level of FAS and FASL proteins compared to control treatment (Figures 2(a), 2(b), and 2(c)). We quantified the activity of caspase-3 by examining the proportion of cleaved-caspase-3 fragment (17 kDa) in the FL-caspase-3 (35 kDa). Compared with the wild control group, isoflurane significantly increased the activity of caspase-3 in the hippocampus of wild type neonatal mice (Figures 2(d) and 2(e); *P* < 0.05, *n* = 5).

### 3.3. FAS or FASL Knockout Attenuates the Increase of Caspase-3 Induced by Isoflurane

**Figure 3:**
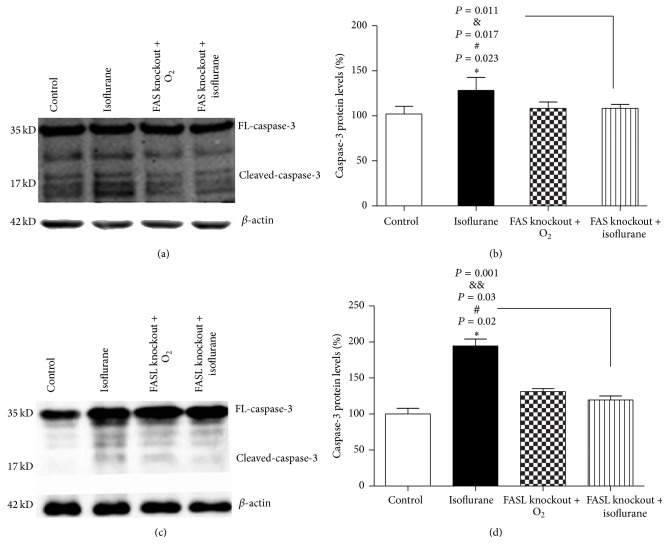
(a) Western blot showed the caspase-3 expression of the four groups. FAS-knockout mice had comparable baseline caspase-3 levels with wild type control mice. Treating FAS-knockout mice with isoflurane did not increase caspase-3 levels (*n* = 5). Wild isoflurane mice showed the highest expression of caspase-3. (b) Quantification of the western blot for caspase-3 levels showed that isoflurane anesthesia caused an increase in caspase-3 level in hippocampus of wild mice compared with FAS-knockout mice plus oxygen group (^*∗*^ *P* = 0.023). FAS knockout reduced the level of caspase-3 caused by isoflurane (^#^ *P* = 0.017). At last, there was an interaction between FAS knockout and isoflurane anesthesia in that FAS knockout mitigated the isoflurane-induced increase in caspase-3 levels in the hippocampus of neonatal mice (^&^ *P* = 0.011). No differences in caspase-3 expression were found among wild type control mice, FAS-knockout control mice, and FAS-knockout isoflurane mice. (c) FASL-knockout mice had comparable baseline caspase-3 levels with wild type control mice. Treating FASL-knockout mice with isoflurane did not increase caspase-3 levels (*n* = 5). Wild isoflurane mice showed the highest expression of caspase-3. (d) Quantification of the western blot for caspase-3 levels showed that isoflurane anesthesia caused an increase in caspase-3 level in hippocampus of wild mice compared with FASL-knockout mice plus oxygen group (^*∗*^ *P* = 0.02). FASL knockout reduced the level of caspase-3 caused by isoflurane (^#^ *P* = 0.03). At last, there was an interaction between FASL knockout and isoflurane anesthesia in that FASL knockout mitigated the isoflurane-induced increase in caspase-3 levels in the hippocampus of neonatal mice (^&&^ *P* = 0.001). No differences in caspase-3 expression were found among wild type control mice, FASL-knockout control mice, and FASL-knockout isoflurane mice. Error bars represent the standard deviation (SD) of four independent experiments; *P* < 0.05.

The homozygous FAS- and FASL-knockout mice (B6.MRL-Fas^lpr^ and B6Smn.C3-Fasl^gld^, resp.) are mice that have a nonfunctional mutation in the FAS gene and FASL gene, respectively [18, 19]. The expression levels of caspase-3 protein in the hippocampus of FAS-knockout mice treated with pure oxygen or isoflurane were both comparable to wild type control mice. However, the expression of caspase-3 in wild type mice treated with isoflurane was clearly increased compared with FAS-knockout mice treated with pure oxygen or isoflurane, *P* < 0.05. The result of two-way ANOVA suggested that loss of FAS attenuates the increase of caspase-3 induced by isoflurane (Figures 3(a) and 3(b)).

We found similar results using FASL-knockout mice. There was no significant difference in the level of caspase-3 between the wild type control group and FASL-knockout mice treated with pure oxygen or isoflurane. However, caspase-3 levels were decreased in FASL-knockout mice treated with pure oxygen or isoflurane compared with the wild type isoflurane-treated group. Two-way ANOVA revealed that loss of FASL attenuates the increase of caspase-3 induced by isoflurane (Figures 3(c) and 3(d)). Together, our results suggested that isoflurane activated caspase-3 through the FAS-FASL pathway.

### 3.4. Isoflurane Increases TUNEL-Positive Cells in the Hippocampus of Wild Type Neonatal Mice but FAS or FASL Knockout Attenuates the Increase of TUNEL-Positive Cells Induced by Isoflurane

**Figure 4:**
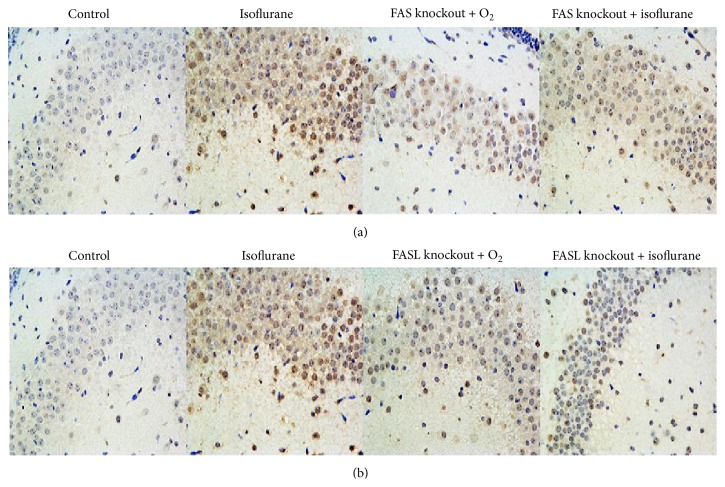
(a) Isoflurane increased the number of TUNEL-positive cells in the hippocampi of wild type neonatal mice compared with wild type control mice. FAS knockout had no effect on baseline TUNEL staining. Isoflurane did not increase TUNEL-positive cells in FAS-knockout mice compared to wild type isoflurane-treated mice. The scale bar represents 50 *μ*m. (b) FASL knockout had no effect on baseline TUNEL staining. Isoflurane did not increase TUNEL-positive cells in FASL-knockout mice compared to wild type isoflurane-treated mice. The scale bar represents 50 *μ*m.

We also determined the degree of neuronal apoptosis by counting TUNEL-positive cells in the hippocampus of mice exposed to isoflurane or oxygen treatment. Isoflurane significantly increased the number of TUNEL-positive cells in wild type mice compared to the wild control group (Figure 4). However, loss of either FAS or FASL occluded this isoflurane-dependent cell death in the hippocampus (Figures 4(a) and 4(b)). FAS/FASL-gene-knockout mice showed fewer apoptosis cells in hippocampus no matter whether they were in pure oxygen group or isoflurane group compared to wild isoflurane group.

### 3.5. Isoflurane Impairs Performance in the MWM of Wild Type Neonatal Mice but Not FAS- and FASL-Knockout Neonatal Mice

**Figure 5:**
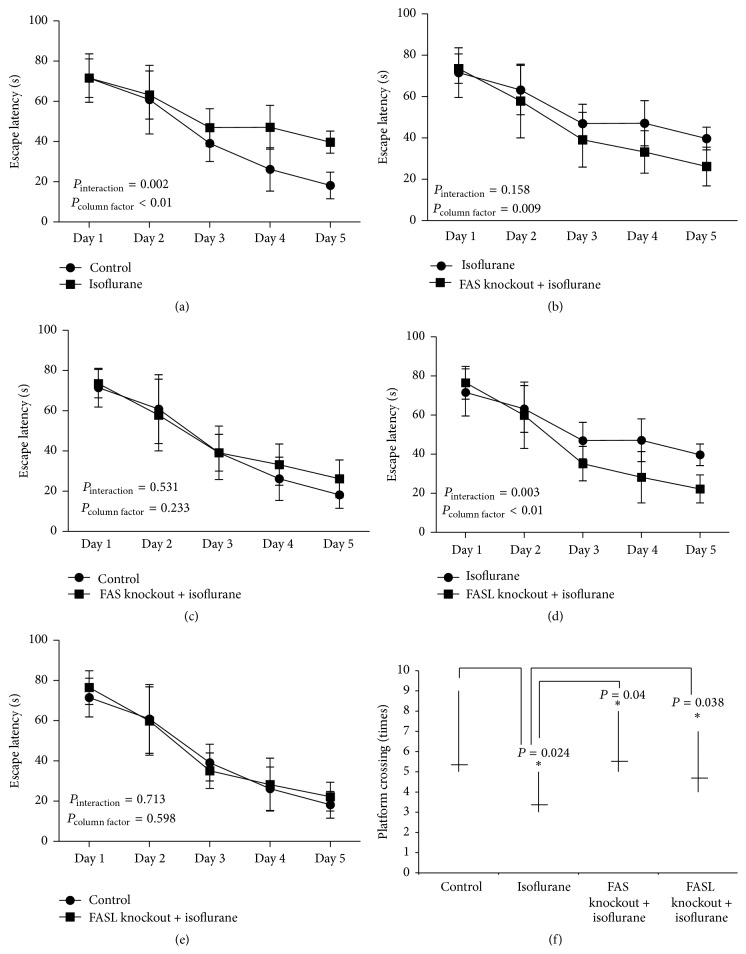
(a) Isoflurane prolonged the escape latency (EL) of wild type neonatal mice compared with the wild type control group. The effects of time and group differences between the two groups were significant; *P* < 0.05. (b and d) Isoflurane did not prolong the EL of either FAS-knockout or FASL-knockout mice compared with wild type neonatal mice treated with isoflurane. The effects of time and group differences between the two groups were significant. (c and e) EL of the wild type control group and isoflurane-treated FAS- and FASL-knockout mice were not significantly different. (f) Isoflurane significantly reduced the platform crossing times of wild type neonatal mice compared with wild type control mice and isoflurane-treated FAS- and FASL-knockout mice.

The MWM was used to study any hippocampal-dependent learning and memory changes elicited by isoflurane. The escape latency of wild type mice was clearly prolonged after isoflurane treatment (Figure 5(a)). There was no significant difference in escape latency between the FAS- and FASL-knockout mice with isoflurane anesthesia and the wild type control mice (Figures 5(c) and 5(e)). However, the escape latency was drastically shortened in the FAS- and FASL-knockout mice treated with isoflurane compared with the wild type isoflurane group (Figures 5(b) and 5(d)). Two-way ANOVA showed that loss of either FAS or FASL attenuates the prolongation of escape latency induced by isoflurane. Comparing platform crossings among the four groups (wild type control group, wild type isoflurane group, and FAS- and FASL-knockout mice treated with isoflurane) showed that wild type mice treated with isoflurane had fewer crossings compared with the other three groups (Figure 5(f)).

## 4. Discussion

Isoflurane is a commonly used clinical anesthetic, and millions of children worldwide that undergo surgery are anesthetized with isoflurane for prolonged time periods that may induce subsequent learning and memory deficits [1, 4, 5]. Thus, we used 7-day-old mice to model clinical anesthesia in young children to explore how isoflurane can affect brain development together with learning and memory. Our findings show that isoflurane induces FASL-FAS-dependent neuronal death, impairing hippocampal-dependent learning and memory.

Isoflurane exposure during early development could cause apoptosis in the developing hippocampus and subsequent memory and learning disabilities. Indeed, recent work showed that isoflurane exposure during early postnatal development resulted in an increase in apoptosis and subsequent behavioral impairment [5, 20]. However, while previous work has proposed some potential upstream mechanisms, other possibilities have not been ruled out. Initially, using genome-wide mRNA profiling of primary cortical neurons treated with isoflurane, we found evidence indicating that isoflurane activates FASL-FAS signaling. Specifically, we found that isoflurane treatment upregulates genes coding apoptosis-related proteins and downregulates those coding learning and memory related proteins. By investigating apoptotic cell markers, we found that isoflurane treatment of wild type mice, but not FAS- or FASL-knockout mice, induced apoptosis, strongly implicating the FASL-FAS signaling pathway in this apoptosis, confirmed by TUNEL staining. Importantly, isoflurane treatment resulted in fewer dead neurons in FAS- or FASL-knockout mice. Behaviorally, we found that wild type mice treated with isoflurane had longer escape latencies in the MWM compared to isoflurane-treated FAS- or FASL-knockout mice. Taken together, we provide a novel mechanism of isoflurane-induced cell death.

FAS (also known as APO-1 or CD95) belongs to a subset of the TNF-R family that is involved in death transducing signals and can trigger apoptosis of neurons. Its physiological ligand, FASL (CD95L), is a member of the corresponding TNF cytokine family [11]. FAS expression can be boosted by cytokines such as interferon-*α* and TNF but also by the activation of lymphocytes [21]. FAS-mediated apoptosis is triggered by its cognate ligand, FASL, which is a TNF-related type II transmembrane molecule and expressed in a far more restricted way than the receptor. Killer cells (the so-called cytotoxic T lymphocytes) remove, for example, virus-infected cells, and those that express FASL can do so by interacting with the FAS receptor on their targets [22]. Isoflurane could increase the expression of FAS and FASL protein in neurons to activate the signaling pathway inducing apoptosis in the hippocampus. Because of its central role in learning and memory, it is not surprising that loss of hippocampal neurons resulting from isoflurane treatment compromises learning and memory. Previous studies have shown that FASL-FAS-dependent neuronal apoptosis plays a critical role in the mechanisms underlying the function of various medicines and substances [12–14]. Our current study, by showing that FASL-FAS signaling activated by isoflurane damages the developing mouse hippocampus, adds to this growing literature. However, the mechanism of how isoflurane promotes the expression and activity of FASL and FAS still needs further researches. Future work should also elucidate whether other commonly used anesthetics also produce this unwanted caveat. Although we did not examine the effects of FAS and FASL knockout on neuronal development, previous work has shown that loss of either gene has no effect on neuronal development [23, 24].

In conclusion, clinically relevant isoflurane anesthesia can damage the developing mouse hippocampus by FASL-FAS signaling. So we should avoid using isoflurane anesthesia in children in clinic to reduce the harm in developing brain. We may also avoid the damage caused by isoflurane to children by exploring the blocker of FASL-FAS signaling. Our study provides a new direction for clinical research in reducing isoflurane-induced damage in children's brains.
